# A spatial epidemiological analysis of self-rated mental health in the slums of Dhaka

**DOI:** 10.1186/1476-072X-10-36

**Published:** 2011-05-20

**Authors:** Oliver Gruebner, Md Mobarak H Khan, Sven Lautenbach, Daniel Müller, Alexander Kraemer, Tobia Lakes, Patrick Hostert

**Affiliations:** 1 Geomatics Lab, Geography Department, Humboldt-Universität zu Berlin, Germany; 2 Department of Public Health Medicine, University of Bielefeld, Germany; 3 Department for Computational Landscape Ecology, UFZ-Helmholtz Centre for Environmental Research, Germany; 4 Leibniz Institute of Agricultural Development in Central and Eastern Europe (IAMO), Germany

## Abstract

**Background:**

The deprived physical environments present in slums are well-known to have adverse health effects on their residents. However, little is known about the health effects of the social environments in slums. Moreover, neighbourhood quantitative spatial analyses of the mental health status of slum residents are still rare. The aim of this paper is to study self-rated mental health data in several slums of Dhaka, Bangladesh, by accounting for neighbourhood social and physical associations using spatial statistics. We hypothesised that mental health would show a significant spatial pattern in different population groups, and that the spatial patterns would relate to spatially-correlated health-determining factors (HDF).

**Methods:**

We applied a spatial epidemiological approach, including non-spatial ANOVA/ANCOVA, as well as global and local univariate and bivariate Moran's *I *statistics. The WHO-5 Well-being Index was used as a measure of self-rated mental health.

**Results:**

We found that poor mental health (WHO-5 scores < 13) among the adult population (age ≥15) was prevalent in all slum settlements. We detected spatially autocorrelated WHO-5 scores (i.e., spatial clusters of poor and good mental health among different population groups). Further, we detected spatial associations between mental health and housing quality, sanitation, income generation, environmental health knowledge, education, age, gender, flood non-affectedness, and selected properties of the natural environment.

**Conclusions:**

Spatial patterns of mental health were detected and could be partly explained by spatially correlated HDF. We thereby showed that the socio-physical neighbourhood was significantly associated with health status, i.e., mental health at one location was spatially dependent on the mental health and HDF prevalent at neighbouring locations. Furthermore, the spatial patterns point to severe health disparities both within and between the slums. In addition to examining health outcomes, the methodology used here is also applicable to residuals of regression models, such as helping to avoid violating the assumption of data independence that underlies many statistical approaches. We assume that similar spatial structures can be found in other studies focussing on neighbourhood effects on health, and therefore argue for a more widespread incorporation of spatial statistics in epidemiological studies.

## Background

The number of people living in slum communities has been increasing rapidly in the megacities of developing countries over the past 20 years [1]. Indeed, although there are already at least one billion people living in slums today, it is projected that this number will double by 2030 [2]. Dhaka, the capital of Bangladesh, is one of the fastest growing megacities in the world and in 2005, approximately 3.4 million out of the city's 12.6 million inhabitants were living in slums [3,4]. Today, the city comprises approximately 14 million inhabitants [4] with more than 300,000 new migrants, mainly the rural poor, moving to Dhaka each year [5,6]. Most of these new immigrants initially concentrate in slums [7,8].

Slum settlements do not provide satisfactory infrastructures such as adequate water supply or sanitary facilities. Overcrowding, non-durability of housing, and insecurity of tenure are common in these settlements [1,9,10]. To accomplish Target 11 of the United Nations' Millennium Development Goals (namely, by 2020 to have achieved a significant improvement in the lives of at least 100 million slum dwellers) [11], public health risk assessments are urgently required. Such assessments need to consider the slum residents' specific health problems that are related to the specific unhealthy socio-physical environments in and around slums. While the aetiology of diseases related to physical environments (e.g., cholera due to poor sanitation facilities) is well understood, health-promoting (or damaging) neighbourhood social and physical effects in slums are not [12,13]. In addition to studying diseases and symptoms, research on the mental health of urban slum populations is also of major importance, as mental and physical health complements each other [1,14,15]. The results of such assessments will support slum-upgrading strategies.

Epidemiological studies concerning public health in Bangladesh and many other developing countries have so far concentrated on comparing population groups (e.g., residents of a slum community with residents of an affluent urban area). Izutsu et al. [16] compared the quality of life, mental health, and nutritional status of adolescents from non-slum areas with those of adolescents from Dhaka's slums. Not surprisingly, slums were associated with worse physical environment and poorer quality of life, as well as gender- and area-specific mental health problems when compared with non-slums. Khan et al. [17] found that socioeconomic characteristics, access to basic and health care facilities, and housing and physical environmental characteristics differed remarkably among urban slums, the urban affluent, and rural areas in and around Dhaka. A higher level of self-rated poor health status was reported by inhabitants living in slums and rural areas.

To our knowledge, no previous study has analysed (i) the associations of neighbourhood socio-physical characteristics with mental health, and (ii) the spatial variation of mental health in urban slums of developing countries. Therefore, we aimed to fill these gaps by investigating mental well-being in selected slums of Dhaka. One well-established approach for assessing the variation of mental health is the WHO-5 Well-being Index [18], which is a quick, reliable, and valid means for assessing psychological well-being or depression [19-22]. The index is quick because it contains only five questions, which are fewer than other tools such as the Beck Depression Inventory, comprised of 21 questions [23], the Centre for Epidemiological Studies Depression Scale (CES-D) [24] with 20 items, the General Health Questionnaire, composed of 12 questions (GHQ-12), or the Patient Health Questionnaire, composed of 9 questions (PHQ-9) [25]. The WHO-5 was successfully applied in both developed [25-28] and developing countries [19,29,30]. Although the WHO-5 was not yet validated in Bangladesh, it was found reliable and effective among elderly Indian communities [19], which are socioeconomically similar to Bangladeshi communities.

A spatial approach to mental health with a focus on spatial structures and autocorrelation of the data prevents a violation of the assumption of data independence, biased coefficient estimates, and p-values biased towards rejecting the null-hypothesis. Furthermore, a spatial approach to epidemiological data leads to the spatial estimation and presentation of health outcomes with the aim of assessing health inequalities, generating hypotheses, and estimating spatial variability in the underlying risks for poor health [31]. Spatial epidemiological analysis also enhances well-established techniques such as regression analysis by explicitly addressing autocorrelation. In recent years, spatial statistics have been increasingly used for quantifying and assessing variations in health status in a variety of studies. For example, Demirel et al. [32] used a spatial autocorrelation analysis to identify locations with high disease rates in Turkey; Pouliou and Elliott [33] detected spatial clusters of overweight and obese populations in Canada; and Sugumaran et al. [34] used the Anselin Local Moran's *I *statistic to uncover spatial clusters of human West Nile virus incidence at the county level in the continental United States.

Such spatial analysis focuses on trends and factors on a specific spatial scale, which, in our case, is embedded in the urban context. Since mental health and physical health are both interrelated [15] and frameworks for mental health in urban slums are rare, we based on a framework for urban health proposed by Gruebner et al. [35], in order to interpret our findings. The framework assumes that the urban context is defined by both physical and social environments that ultimately influence urban health across all scale levels. The health disparities of residents living in distinct neighbourhoods within slums may be due to varying socio-physical effects between these neighbourhoods (e.g. neighbourhood socio-economic status or environmental quality) and the slum residents' personal resources (e.g., personal socio-economic status or housing quality). See Diez-Roux and Mair [12] for evidence from non-slum neighbourhoods. The goal of this study was to investigate the spatial variability of self-rated mental health status for different population groups in several slums of Dhaka, focussing on the individual, household, and neighbourhood levels. We investigated the hypotheses that mental health shows a significant spatial pattern (e.g., spatial clustering) for different population groups, and that the spatial patterns relate to spatially-correlated health-determining factors (HDF), for example, housing quality or income generation ability.

This study dealt not only with new research questions that are very important from a public health point of view, but also combined two well-established methods from two different disciplines. Moreover, we could generalise our findings in similar settings of developing countries. Briefly, our spatial epidemiological approach will enhance our understanding of specific factors contributing to the health of slum residents.

## Methods

### Study design and variables

**Figure 1:**
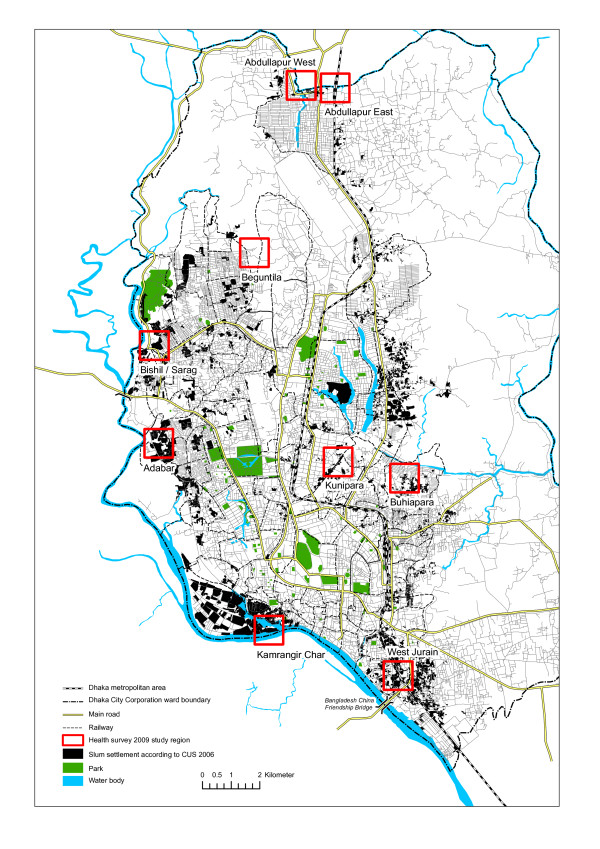
**Dhaka City, cohort study (2009) and corresponding slum settlements**.

From March to April 2009, we conducted a cohort study in nine Dhaka slum settlements (figure 1). In total, 1,938 slum household members were interviewed face-to-face by trained university graduates. Before starting the interview, the aims of the survey were explained and verbal consent from each respondent was given. Although various types of information were collected, only some relevant variables from the baseline data were used for this study. We also used global positioning system (GPS) devices to record the location of each household interviewed.

The WHO-5 was used as a measure of self-rated mental health status. The brief screening instrument assessed the indicators of depression by five questions rated on a 6-point forced-choice Likert scale [36], from 0 to 5. The rates were summed to a range from 0 to 25. Within that range, a raw score of < 13 suggested poor well-being. Please refer to [18] for more details on the WHO-5 questionnaire.

Other self-rated health outcomes were collected to test for the reliability of the WHO-5. For "self-rated health", we asked respondents how they would generally rate their health, with the possible answers being "poor" (coded as 1), "fair (2)", "so-so" (3), "good" (4), and "excellent" (5). As a third measure for health status, we asked whether respondents had suffered from any disease in the three months prior to the survey, coded as 0 for yes and 1 for no. Generally, we coded all categorical variables so that the higher values hypothetically indicate a better health-related quality of life.

Explanatory covariates for the neighbourhood, household, and individual levels were taken from the abovementioned survey and from satellite data analysis. We extracted 14 principal components from these covariates to represent the socio-physical environment and individual health knowledge and behaviour (table 1). The 14 identified health-determining factors (HDF) explained 59.5% of the variance in the data, ranging from 6.3% (housing quality) to 3.4% (personal health knowledge). Another 7 HDF were considered independent from the 14 principal components: community membership, bed net usage, education, marital status, migration background, age, and gender.

**Table 1:** Components of health-determining factors (HDF) used in this study

**Housing quality** A higher monthly rent for the house (0.6), owning a gas burner (0.8), better construction materials (0.5), and also better-quality fuel for cooking (0.9) contributed to a higher quality of housing. Hence, the first component was named 'housing quality', which explained 6.3% of the variance.
**Population density** 'Population density' explained 5.2% of the variance and was constituted through a higher number of family members (0.8), a higher number of persons sharing the same meals (0.7), and a higher number of persons living in the same room (0.7).
**Smoking behaviour** Not smoking cigarettes (0.8), not smoking inside the room (0.8), and a small number of family members who smoke (-0.7) were correlated with the component 'smoking behaviour', which explained 4.8% of the variance.
**Access to basic services** A large distance to the nearest park area (-0.8), a short distance to the nearest river (0.5), owning an electric fan (0.6) and a better water supply (0.5) correlated with this component, which we named 'access to basic services'. Owning an electric fan was regarded as a substitute for having an energy supply. This component explained 4.7% of the variance.
**Household wealth** This component was correlated with owning a radio (0.6), owning a TV (0.6), owning a tape/CD/VCD (0.7) and the number of rooms (0.5). Having more rooms increases, for example, the capacity to generate income by renting out extra rooms. All of these variables indicate the household's material wealth, so the component was named accordingly. It explained 4.3% of the variance. Note that e.g., the wealth index used by Rutstein and Johnson [66] contained more factors than that of our study.
**Natural environment** Larger amounts of vegetation 100 metres around the households (0.8), lesser amounts of surface water 100 metres around the households (-0.6) and longer distances to the nearest major street (0.7) were correlated with the component 'natural environment', which explained 4.3% of the variance.
**Flood non-affectedness** Longer distances to the nearest river (0.5), whether the area was regarded as flood non-affected (0.7), and whether the area had an adequate drainage system (0.7) were correlated with this component, which explained 4.1% of the variance in the data.
**Job satisfaction** Fewer working hours (-0.4), liking one's job (0.7), and not thinking that the job is harmful to one's health (0.8) were correlated with the component 'job satisfaction', which explained 4% of the variance.
**Environmental health knowledge** Thinking that polluted, stagnant water and garbage near one's house could spread disease and increase the risk of poor health (0.8) and that air pollution is bad for one's health (0.6) were found to be correlated with this component. We named it 'environmental health knowledge', as it reflects awareness that the environment can affect one's health. This component explained 3.9% of the variance.
**Income generation** A larger number of family members earning income (0.7), higher monthly family income (0.7), having a job contract (0.4), and working more hours a day (0.2) were correlated with the component 'income generation'. This component explained 3.7% of the variance.
**Sanitation** A better toilet facility (0.7) and a better garbage disposal attitude (0.6) were correlated with the component 'sanitation', which explained 3.6% of the variance.
**Housing sufficiency** 'Housing sufficiency' was correlated with sufficient light in the house (0.6), whether the room was used for other purposes aside from living (0.7), and whether the room was regarded as sufficient for one's family (0.5). This component explained 3.6% of the variance.
**Housing durability** Two variables, namely the household had a refrigerator (0.7) and the house was considered to be permanent (0.8) were correlated with a component called 'housing durability', which explained 3.5% of the variance.
**Personal health knowledge** 'Personal health knowledge' reflected awareness of personal behaviour and related health effects. Thinking that smoking tobacco is bad for one's health (0.7) and that physical exercise can be good for one's health (0.7) was correlated with this component. It explained 3.4% of the variance.

Health-determining factors (HDF) and their correlated original variables were obtained from a principal component analysis (PCA). More details are available from the authors.

### Sampling strategy for slums

Approximately 4,900 slum settlements in Dhaka were identified by the Centre for Urban Studies (CUS) in 2005 [3]. We established minimum threshold values of 500 households and six acres per slum to select comparable slum settlements from the CUS survey. To achieve an adequate geographical distribution of the slum settlements, we subsequently selected administrative units that usually did not neighbour each other. In units with more than one slum, we randomly selected one of these settlements. We adapted our selection to account for slums in which the residents were evicted, or those converted into affluent residential areas or open spaces since the CUS survey in 2005.

### Sampling strategy for participants

To calculate the minimum sample size *N *[equations 1 and 2, 37] needed to gain a representative sample, we estimated the number of families within each of the slum settlements (*n*_pop_) with the help of local residents and community leaders who verified these values, such that(1)

*n*_inf _= sample size based on the assumption of an infinite background population,

*q *= quantile value of the normal distribution for the selected confidence level,

*p *= probability of selecting an individual with a certain health status (e.g., WHO-5 score < 13),

*d *= acceptable margin of error as a percentage, and(2)

*N *= sample size (for a finite population), and

*n*_pop _= size of the population.

In our study we used a 95% confidence level and an error margin of *d *= 6%. Because it was not possible to run a pilot study to determine an estimate for *p*, we used a 50% probability of choosing the "right" individual (*p *= 0.5). To calculate the sampling rate *r*, we divided the number of families in the slum by the sample size. We then interviewed every *r*^*th *^household. When it was not possible to identify an interview partner at a household, we proceeded to the subsequent one and thereby achieved the target sample size.

### Spatial statistics for epidemiological studies

**Figure 2:**
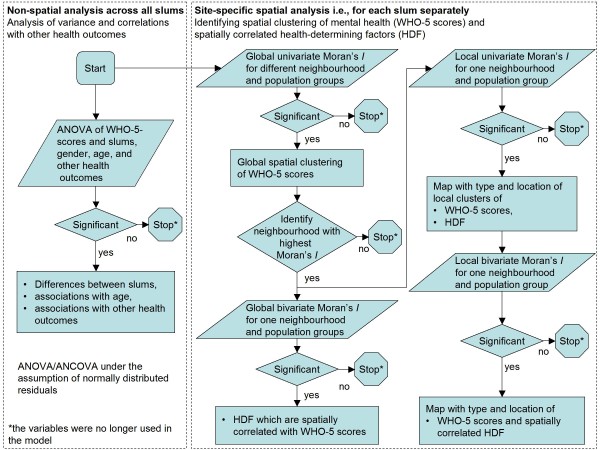
**Spatial epidemiological approach used for this study**. Parallelograms stand for statistical processes, rhombuses for selection criteria and rectangles for outcomes.

We used a spatial epidemiological approach to detect and explain spatial clusters of WHO-5 scores of urban slum residents (cf. figure 2). We started with a (non-spatial) analysis of variance/covariance (ANOVA/ANCOVA) across all slums to identify differences between the slums and to find correlations of WHO-5 scores with age, gender, and other self-rated health outcomes. All subsequent spatial analyses were performed for each slum separately. We focussed on adult slum residents (age ≥15 years) and separated the sampled population in two ways. For the first, we separated the sample population into female and male groups, and for the second, we separated sampled slum residents by age - young adults (15-44 years), middle-aged adults (45-64 years), and older adults (65 and more years) - classified according to the WHO [38]. In the slum-specific spatial analysis, we dropped the older-adult age group because of its small sample size.

Spatial autocorrelation analysis was applied to summarise the degree to which persons with a similar health status tend to occur next to each other (i.e., form spatial clusters) [39]. We therefore concentrated on global clustering rather than on globally-dispersed patterns. Multivariate spatial correlation analysis was used to gain information on the extent to which values for the well-being of one person (*z*_*f*_) observed at a given location show a systematic (more than likely under spatial randomness) association with another variable (*z*_*g*_) observed at the "neighbouring" locations. This multivariate spatial correlation can be considered in addition to or instead of the usual (non-spatial) correlation between two variables at the same location [40].

Spatial autocorrelation statistics depend on the definition of neighbourhood relationships through which the spatial configuration of the sampled subpopulation was defined prior to analysis. Because they can influence the results [41], we explored various neighbourhood definitions. First, we used 30-, 60- and 90-meter fixed-band definitions, which treat every observation point inside that search radius as a neighbour. Second, we used the k-neighbour approach, which treats the nearest k observations as neighbours. We used k values of 3, 5, and 10. For both approaches, we used a binary weight matrix to assign weights to the neighbours. This binary weight matrix assigns a weight of unity for neighbours and zero for non-neighbours. The spatial patterns were investigated by global measures that allowed for spatial clustering tests. For example, in cases of positive spatial autocorrelation, spatially clustered patterns point to the attraction and prevalence of either poor or good well-being. Local indicators of spatial association were applied to indicate the type (e.g., well-being or housing quality, described as either poor or good) and locations of clusters within the settlements [42]. All spatial analyses were performed in GeoDa [43].

#### Global univariate spatial autocorrelation

We applied Moran's *I *[44,45] to account for the global spatial autocorrelation of similar and dissimilar WHO-5 scores of the nine slums of Dhaka. For the Moran's *I *statistic, the sum of covariations between the sites for the distance *d(i,j) *was divided by the overall number of sites *W(d*_*i,j*_*) *within the distance class *d(i,j)*. Thus, the spatial autocorrelation coefficient for a distance class *d*(*i*,*j*) was the average value of spatial autocorrelation at that distance. The Moran's *I *statistic for spatial autocorrelation is defined as follows [46]:(3)

*n *= the sample size,

 row-standardised spatial weights matrix of sites *i *and *j*,

 = sum of the number of sampling locations per distance class,

y_i _= the value at site *i *(e.g., the WHO-5 scores).

The actual value for Moran's *I *was then compared with the expected value under the assumption of complete randomisation:(4)

Moran's *I *values may range from -1 (dispersed) to +1 (clustered). A Moran's *I *value of 0 suggests complete spatial randomness. To verify that the value of Moran's *I *was significantly different from the expected value, we applied a Monte Carlo randomisation test with 9,999 permutations to achieve highly significant values. Data values were reassigned among the N locations, providing a randomised distribution against which one may judge the observed value. If the observed value of *I *was within the tails of this distribution, there was significant spatial autocorrelation in the data, and the assumption of independence among the observations could be rejected [47]. We then selected for the mental health outcomes for each population group and chose from the nine slums the neighbourhood with the highest Moran's *I *for further bivariate analysis.

#### Global bivariate spatial correlation

A bivariate coefficient of spatial correlation between two standardised random variables y_*k *_and *y*_o _is defined as follows [48]:(5)

where  and  have been standardised such that the mean is zero and the standard deviation equals one, and W^s ^is a doubly-standardised spatial weight matrix as described above. Multivariate spatial correlation thus focuses on the extent to which values for one variable y_k _observed at a given location show an association with another variable y_o _observed at the neighbouring locations [40]. This yields the following multivariate counterpart of a Moran-like spatial autocorrelation statistic [40]:(6)

In this manner, we tested the 21 HDF for spatial correlations with mental health. The significance of this bivariate spatial correlation was assessed, as in the univariate case, by means of a randomisation approach [42].

#### Local univariate spatial autocorrelation

We then calculated the local univariate Moran's *I *for WHO-5 scores (and also for HDF) with the identified best neighbourhood (as described above) and respective population group. This allowed us to implement global measures that allow for spatial patterning tests over the whole study region, which test for statistically significant local spatial clusters, including the type and location of these clusters. We concentrated on the Anselin Local Moran's *I *statistic, which is calculated as follows [41,42]:(7)

*W*_*ij*_(*d*) is the row-standardised weights matrix given a local neighbourhood search of radius *d*. The neighbourhood definitions were the same as the global statistics that were applied (i.e., distance and k-neighbours). Assuming complete randomisation, the expected value of *E*(*I*_*i*_) is as follows [46]:(8)

Unlike the global Moran's *I*, which has the same expected value for the entire study area, the expected value of local Moran's *I *varies for each sampling location because it is calculated in relation to its particular set of neighbours [42]. We calculated the significance of the local Moran's *I *using a randomisation test on the Z-score with 9,999 permutations to achieve highly significant values [46]:(9)

Positive spatial autocorrelation occurs when, for example, WHO-5 scores of residents living in one location are surrounded by similar WHO-5 scores of other residents in neighbouring locations (low-low - LL, high-high - HH), thus forming a spatial cluster. Negative spatial autocorrelation appears when high WHO-5 scores are surrounded by low WHO-5 scores (HL) and vice versa (LH) (i.e., when spatial outliers occur) [42]. Because equation 5 does not indicate whether a cluster consists of high or low values, the original WHO-5 scores at the sample points are used for classification into LL, HH, LH or HL.

#### Local bivariate spatial correlation

In a next step, we calculated the bivariate Moran's *I *for the WHO-5 scores (and also HDF) for the best neighbourhood and population group. Using a similar rationale as in the original development of local indicators of spatial association (LISA) [42], the numerator in equation (6) can be decomposed into the contributions of the individual observations [40]. For the traditional univariate Moran's *I *autocorrelation statistic, the local version was termed a local Moran's statistic; its multivariate generalisation can be defined as follows [40]:(10)

using the same notation as before. This statistic provides an indication of the degree of linear association (positive or negative) between the values for one variable y_k _at a given location i, and the average of another variable y_o _at neighbouring locations j, . A greater than indicated similarity under spatial randomness suggests a spatially similar cluster in the two variables. A dissimilarity greater than spatial randomness would imply a strong, local, negative relationship between the two variables [40]. The significance of the statistic was assessed by means of the permutation approach.

## Results

### Variations in well-being among population groups and slums

We found that poor well-being (WHO-5 scores < 13) among the adult population (age ≥ 15 years) was predominant in all slums. Ignoring spatial structures and assuming normally distributed errors, an ANOVA/ANCOVA analysis showed that the WHO-5 scores for males and females did not significantly differ (p = 0.21), but age had a significant negative effect (regression coefficient of -0.06 per year of age, p < 0.001).

**Figure 3:**
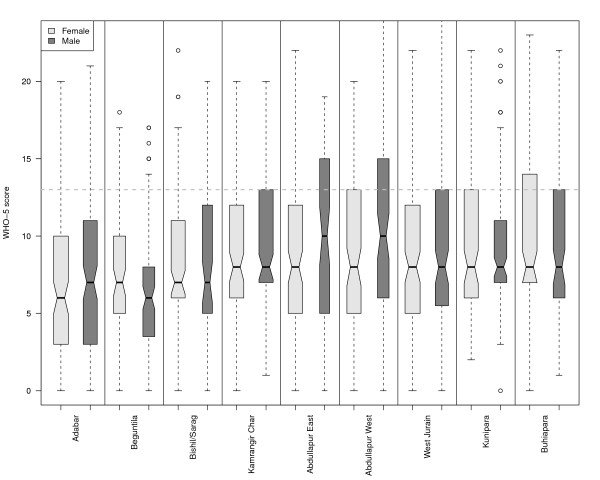
**Box plot for gender groups across slums**.

**Figure 4:**
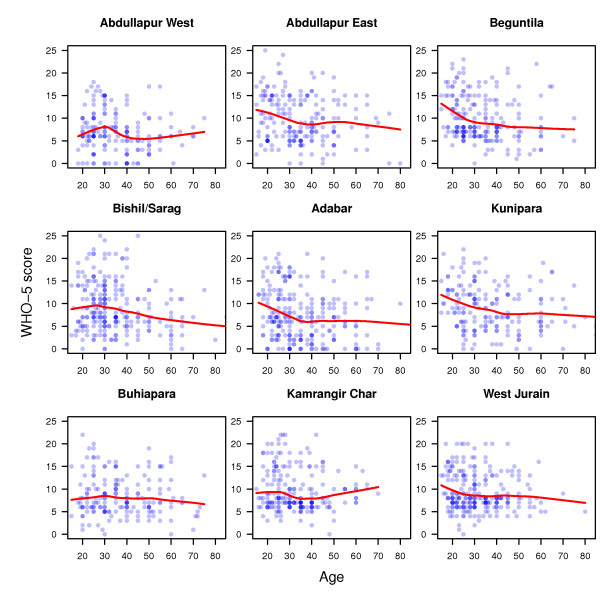
**Co plot for age across slums**.

WHO-5 scores were positively related with self-rated health (regression coefficient 1.99, p < .001) and with 'not having had a disease in the three months preceding the survey' (regression coefficient 1.92, p < 0.001). Furthermore, WHO-5 scores differed significantly between some slums (p < 0.001, cf. figures 3, 4). All predictors had low predictive power. Even the model with the slum, age, and gender predictors explained only 14% of the variance.

### Spatial patterns of well-being

**Figure 5:**
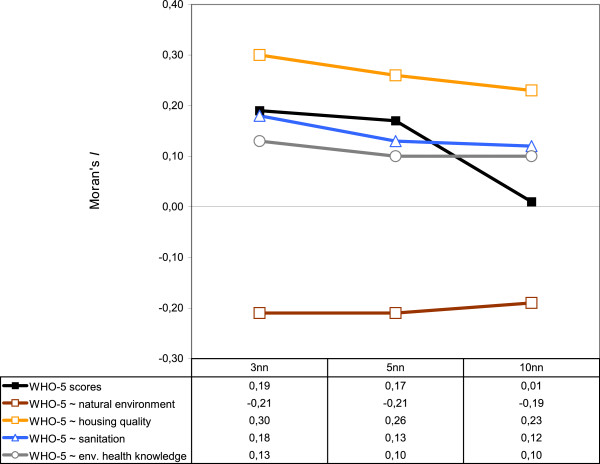
**Moran's *I *values for males in the slum settlement Bishil/Sarag**. Significant global univariate and bivariate Moran's *I *values for mental health (WHO-5 scores) and spatially-correlated health-determining factors are shown for different nearest neighbours. Note that the Moran values decrease as the number of neighbours increases.

We found the strongest global spatial clustering when the three nearest (sampled) neighbours were considered in the analysis (mean distances ranging from 9 to 11.4 meters). Beguntila and Bishil/Sarag were among the settlements with the highest values (cf. table 2). In Beguntila, spatial clustering of well-being was most significant (p < 0.001) among the young adult age group. Within this age group, good well-being was positively associated with housing quality and male gender. Furthermore, we detected a negative association between well-being and 'natural environment' (cf. table 3). In Bishil/Sarag, the strongest and most significant (p < 0.001) global spatial clustering was detected among males. These clusters were positively associated with housing quality, sanitation, and environmental health knowledge; however, they were negatively associated with 'natural environment', a regression factor including the amount of vegetation and water around households, as well as the distances to streets (cf. table 1). To some extent, we also found that spatial clustering among females in Bishil/Sarag was positively associated with flood non-affectedness, housing quality, and education, as well as being negatively associated with age. Within Bishil/Sarag, we also found spatial clustering to be additionally correlated with income generation among young adults. In Abdullapur East and Kunipara, we found spatial clustering of WHO-5 scores only when using a large neighbourhood of the 10 nearest neighbours of respondents (mean distances ranging from 35 to 57 meters). In addition, we found that within slums and within population groups, the strength and significance of spatial autocorrelation differed with the type of neighbourhood relation. For example, spatial autocorrelation among males in Bishil/Sarag decreased when more neighbours or longer distances were considered in the analysis; the same was true among young adults in Beguntila (table 2). Therefore, the global univariate Moran's *I *of the response variable (WHO-5 scores) reflect the spatial variation at the scale of the settlements. Focussing on health-determining factors (HDF) with the global bivariate, Moran's *I *revealed a similar spatial pattern at the scale of the settlements (cf. figure 5).

**Table 2:** Global univariate Moran's *I *values for different neighbourhood relationships

Neighbourhood relationship	Beguntila	Bishil/Sarag				Abdullapur East	Kunipara	Adabar	Buhiapara
	Young adults	Males	Females	Young adults	Middle aged adults	Females	Middle aged adults	Young adults	Total sample
Nearest neighbours									
3 nn	0.16*	0.19**	0.12*	0.12*	.	.	.	.	.
	*(8.5)*	*(11.4)*	*(10.8)*	*(9)*					
5 nn	0.16**	0.17**	.	0.1*	.	.	.	.	.
	*(10.4)*	*(14.7)*		*(11.6)*					
10 nn	0.13**	0.01***	.	.	.	0.06*	0.09*	.	.
	*(14.2)*	*(20.5)*				(35.2)	(57)		
Fixed distance									
30 m	0.1***	.	.	.	.	.	.	.	.
60 m	0.05**	0.13***	.	0.05**	.	.	.	.	.
90 m	.	0.12***	.	0.03*	0.09**	.	.	0.02*	0.02*

Significance levels: < 0.001 '***', < 0.01 '**', < 0.05 '*', > 0.05 '.'

Global Moran's *I *values for those slums and population groups which were significant under a Monte Carlo test with 9,999 permutations (p < 0.05). We only report positive Moran values, i.e., those revealing global spatial clustering. For nearest neighbour-based distances, we report in parentheses the average distance-per-slum in metres. Note that the strongest values occur with three nearest neighbours. We thus used this neighbourhood relationship in the subsequent bivariate Moran's *I *analysis.

**Table 3:** Global bivariate Moran's *I *values for the three nearest neighbours

Scale level	Health-Determining Factor	Beguntila	Bishil/Sarag		
	WHO-5 scores ~	Young adults n = 115	Young adults n = 170	Females n = 104	Males n = 122
**Global univariate Moran's *I *for WHO-5 scores**		0.16*	0.12*	0.12*	0.19**
**Neighbourhood level** physical environment	'Natural Environment' Flood non-affectedness	-0.19*** .	-0.16*** 0.13**	. 0.13*	-0.21** .
**Household level** physical environment	Housing quality Basic services Household wealth Sanitation Housing sufficiency Housing durability	0.13* . . . . .	0.19*** . . . . .	0.14** . . . . .	0.3*** . . 0.18*** . .
**Household level** social environment	Population density Job satisfaction Income generation	. . .	. . 0.1*	. . .	. . .
**Individual level**	Smoking behaviour Environmental HK Personal HK Community member Using bed net Education Married Migrant Age Gender	. . . . . . . . --- 0.12*	. 0.09* . . . . . . --- .	. . . . . 0.11* . . -0.12* ---	. 0.13* . . . . . . . --- .

Significance levels: < 0.001 '***', < 0.01 '**', < 0.05 '*', > 0.05 '.', not applicable '---'

HK: Health knowledge

The table displays health-determining factors that are significantly (p < 0.05) spatially correlated with mental health (WHO-5 scores) of those population groups and slums in which strongest global spatial clustering of WHO-5 scores were found (cf. Table 2). Note that the WHO-5 scores among males in Bishil/Sarag are clustered most strongly, and there is a strong spatial correlation with 'natural environment' and housing quality in this population group.

**Figure 6:**
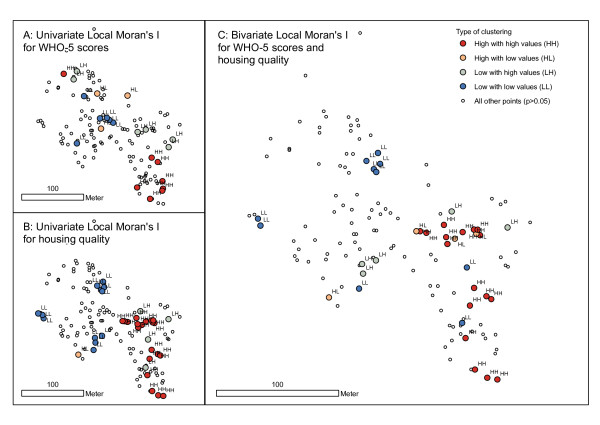
**Local cluster maps for mental health (WHO-5 scores) of males in the slum settlement Bishil/Sarag**. Each dot on the map indicates a slum household (GPS point). The maps indicate significant (p < 0.05) spatial clusters of high (HH) or low (LL) WHO-5 scores (A), housing quality (B), or similar values of both WHO-5 scores and housing quality (C), respectively. High values surrounded by low values (HL) and vice versa (LH) indicate outliers. The three nearest neighbours of a household were used in the statistics.

Local cluster maps derived using Anselin's Local Moran's *I *statistic (local univariate Moran's *I*) [42] were used to calculate the type and location of the clusters detected. Because clustering was strongest and most significant in Bishil/Sarag, we concentrate on this slum in the following analysis. We found that for this settlement, well-being among males was spatially structured in the west and east, with poor well-being localised predominantly in the western area and good well-being in the eastern part of the settlement (cf. figure 6). Furthermore, the local bivariate Moran's *I *statistic revealed that low WHO-5 scores were associated with poor housing quality in the western part of Bishil/Sarag, whereas high WHO-5 scores and better housing quality were clustered in the east. 'Natural environment' was found to be negatively correlated with well-being: in the western area, a higher amount of 'natural environment' could be found, together with poor well-being clusters (results not shown). In contrast to the spatial clustering of males, both patterns of good and poor well-being appeared among females in the western part of Bishil/Sarag (results not shown).

## Discussion

Individual characteristics such as age or gender are associated with health and give rise to a unique spatial structure among the health status of different population groups. Although gender was (surprisingly) not associated with mental health in our non-spatial ANOVA, we investigated spatial structures separately and found different spatial patterns for both groups, which supports our first hypothesis. When defining the three nearest (sampled) neighbours as a neighbourhood, we provided evidence that clustered households/respondents contrast strongly with other households/respondents that are not in that cluster by having better or poorer well-being. Hence, it can be stated that first, there are substantial health inequalities in slums. Secondly, the investigated patterns show a spatial dependence of well-being at one location on the well-being of a neighbouring location. A possible explanation is that good mental health in a neighbourhood may support salutary effects in the social fabric, and vice versa [49]. Focussing on our second hypothesis, our study showed that the association between individual characteristics such as age or gender and mental health status in slums is often indirect and can be heavily influenced by other factors such as neighbourhood socio-physical characteristics. These factors shape the distinct vulnerabilities and the resilience of residents towards ill health. The example of Bishil/Sarag showed that the spatial distribution of poor housing quality was correlated with the spatial distribution of poor well-being and vice versa. Thus, we not only provided evidence that the well-being of slum residents is associated with certain HDF in the same households, but that well-being is also associated with HDF prevalent in the immediate neighbourhood of a respondent's home. In other words, neighbourhood clusters, which constitute significantly better or poorer well-being in comparison to the other households of a slum, also comprise similar HDF that are related to their respective health status.

The rapid urban expansion of Dhaka has facilitated a huge loss of prime agricultural areas and wetlands [50], which are generally known to provide important provisioning and regulation ecosystem goods and services (ESS) that can support health in a variety of ways [14]. Goods and services such as climate regulation, air and water purification, or outdoor recreation [51], can be thought of as being related to physical HDF at the municipal and neighbourhood level [35]. In Dhaka, water retention areas have been increasingly lost due to the widespread practice of earth in-filling while building ground construction. The loss of ESS regulation, combined with poor infrastructural planning, has thus led to deteriorating living conditions and increased environmental risks, particularly the risk of flooding [52]. It is consequently quite understandable that not being affected by flooding was found to be positively associated with mental health in our study.

Having large areas of vegetation in the nearby neighbourhood often increases the health-related quality of life, for example, by reducing heat stress induced through a local urban heat island effect [53-55]. Furthermore, urban green and park areas are typically considered to be recreational facilities for urban residents [56,57]. In Dhaka's slums, vegetation cover is scarce, and we therefore assumed a strongly positive association between nearby green areas and mental health. However, many of those areas that we had expected to improve living conditions and thus mental health, turned out to be low-lying and regularly flooded areas. Combined with poor sanitation, open waste water drainage and garbage disposal, such vegetation patches increase the risk for infectious diseases (e.g., diarrhoea). In Bishil/Sarag we identified an example for a local negative association between high values of 'natural environment' and well-being among males in the western part of the settlement. Bishil/Sarag represents the situation in most slums in Dhaka where the open areas are particularly heavily polluted. Our analysis thus identified environmental disservices rather than services. In contrast, good housing quality is positively correlated with health by reducing the risk of asthma and other respiratory conditions, injuries, and psychological distress, as well as by supporting child development [58]. Likewise, poor sanitation is correlated with the risk of infectious diseases and symptoms; for example, gastrointestinal diseases or respiratory diseases may arise because of factors such as poor ventilation [58]. Exposure to the socio-physical environment is thus determined by socio-economic status (SES), which defines a large part of a household's resilience [59] in response to a health threat [60-62]. However, we could only further identify a spatial association of mental health with income generation ability, but not with household wealth. Within Dhaka's slums and considering spatial dependencies, SES thus seems to be best described by housing quality and sanitation. At the individual level, mental health was positively associated with environmental health knowledge, mainly in Bishil/Sarag. This association may reflect the person's awareness of environmental threats and eventual adaptation strategies. The fact that the spatial clustering of mental health among females was negatively associated with age and positively correlated with education is also in accordance with prior results commonly found in non-spatial analyses [16,62-64].

In our study, the strength of spatial clustering decreased when more of the nearest neighbours or higher distances to the neighbours of respondents were considered for spatial autocorrelation analysis. This was demonstrated by the mental well-being among males in Bishil/Sarag, but could also be revealed by the HDF for this population group. This spatial pattern could also be verified among other population groups within the same slum and in Beguntila. Overall, it can be stated that such spatial patterns point to small-scale effects within the slums, indicating that autocorrelation effects and the spatial effects of HDF take place at short distances. Moreover, our results provide evidence that model outcomes are sensitive to different definitions of neighbourhood relation. Considering that spatial patterns of health status uncover health disparities and provide the basis for further analysis, our study helps to determine the feasibility of using a particular statistical method to avoid violating the assumption of data independence that underlies most non-spatial statistical approaches [39,41,65]. For subsequent analyses on health and the environment in the spatially autocorrelated settlements, any statistical model used has to be extended to account for the particular spatial dependence in the data. Identifying an appropriate neighbourhood relationship for a variety of spatial analysis methods is thereby a crucial endeavour.

### Limitations

The analysis of the spatial autocorrelation of mental health in and between slums was challenging for several reasons. One of the challenges included obtaining a representative sample in Dhaka's slums because of the high population density and poor accessibility of the slum households. It was almost impossible to achieve a geographically well-distributed set of sample points representing all slum residents within the slums. Furthermore, the nine slums selected for the analysis may not fully represent all ~4,900 rather small slum clusters in Dhaka. Second, the WHO-5 Well-being Index had never been tested with slum residents, and validation studies for its reliability are highly recommended. Third, we might have missed some influential HDF in our model (e.g., air pollution, social capital, or accessibility to health care facilities), which would thus result in rather weak spatially-correlated mental health patterns in the slums. Fourth, the bivariate Moran's *I *statistic suggested a linear relationship between well-being and the covariates, which might not mirror the true relationship between the variables. Furthermore, the bivariate model did not control for other effects like gender and age, and interactions between the covariates were not yet incorporated within the model. However, our research presents a method to account for the relationship between health and the environment in a spatial epidemiological model.

## Conclusions

Adding to the existing literature on public health in slums, we were able to contribute empirical evidence for the local variation of well-being in selected slums of Dhaka. We conclude that the WHO-5 Well-being Index is an easy-to-use and quickly assessed measure for mental health in slums. The WHO-5 scores were positively correlated with other health outcomes such as 'self-rated health' and negatively with 'having had a disease'.

When defining the three nearest (sampled) neighbours as a neighbourhood, we provided evidence that identified clusters constitute significantly better or poorer well-being in comparison to the other households of a slum, and further, that these clusters comprise similar neighbourhood socio-physical characteristics that are related to the respective mental health status. Moreover, we provided evidence that spatial dependencies are sensitive towards the spatial relationships i.e., the definition of the neighbourhood under investigation.

Knowledge of the spatial distribution and structure of one's health status helps us to understand a community's social fabric and its related health-determining factors, but most importantly, it allows for a more efficient and effective spatial allocation of scarce resources to target the alleviation of poverty and the improvement of living standards. Because our methodology provides evidence for spatial dependencies in epidemiological data, it might lead to more sophisticated spatial epidemiological models that create a deeper understanding of functional relationships between health and the environment. In addition to examining health outcomes, our methodology can also be adapted to investigate regression residuals and thus help avoid the violation of data independence that underlies many statistical approaches. Spatial epidemiological models could thus lead to improved rationales for public health interventions and might strengthen policy significance. This type of approach is of vital relevance in developing specific strategies for improving the lives of slum dwellers in Dhaka and in comparable settings worldwide.

We argue for a more widespread use of spatial epidemiological approaches in similar public health studies because we assume that our conclusions are relevant for other studies in the slums of developing countries.

## Competing interests

The authors declare that they have no competing interests.

## Authors' contributions

OG carried out the cohort study, designed the paper, performed statistical analyses and drafted the manuscript. MMHK designed and carried out the cohort study and helped interpret the findings. SL and DM participated in the design of the paper and guided the statistical analysis and interpretation. AK and PH obtained the grant, designed the overall framework and coordination, and helped to draft the manuscript. TL helped in designing the manuscript and revised it critically. All authors read and approved the final manuscript.
